# Resilience Mediates the Association Between Discrimination and Psychological Distress Among Rural, Black Women in South Carolina: A Structural Equation Model

**DOI:** 10.1007/s40615-025-02559-7

**Published:** 2025-07-29

**Authors:** Camryn Garrett, Abhishek Aggarwal, Huiyi Xia, Cheuk Chi Tam, Bankole Olatosi, Sara Wilcox, Xiaoming Li, Shan Qiao

**Affiliations:** 1 South Carolina SmartState Center for Healthcare Quality, Arnold School of Public Health, University of South Carolina, Columbia, USA; 2 Department of Health Promotion, Education, and Behavior, Arnold School of Public Health, University of South Carolina, Columbia, USA; 3 Department of Health Services Policy and Management, Arnold School of Public Health, University of South Carolina, Columbia, USA; 4 Department of Exercise Science, Arnold School of Public Health, University of South Carolina, Columbia, USA; 5 Prevention Research Center, Arnold School of Public Health, University of South Carolina, Columbia, USA

**Keywords:** Discrimination, Resilience, Psychological well-being, Structural equation modeling

## Abstract

**Introduction:**

The marginalization of intersecting racial, gender, and rural identities compounds the structural vulnerabilities (e.g., discrimination, stigma, health disparities) experienced by Black women in South Carolina’s rural areas. Discriminatory experiences can deteriorate mental health; however, resilience can potentially reduce the ill effects of discrimination by decreasing associated psychological distress. This study examines the mediating role of resilience in the association between discrimination and psychological distress among rural, Black women in South Carolina in the post-pandemic era.

**Methods:**

Black women (*n* = 300, mean age = 48.6) living in rural South Carolina were recruited from January to February 2024 using a cluster sampling method. Participants completed surveys assessing experiences of discrimination, resilience, and psychological distress (i.e., anxiety, depression, PTSD). A structural equation modeling analysis was performed using the Lavaan package in R.

**Results:**

Discrimination was negatively associated with resilience (*β* = − 0.107, *p* < .05) and positively associated with psychological distress (*β* = 0.387, *p* < .001). Discrimination had a reduced, positive indirect effect on psychological distress through the path of resilience (*β* = 0.042, *z* = 2.004, *p* < .05), after controlling for participants’ demographics. The model fit was satisfactory (RMSEA = 0.038; CFI = 0.986; TLI = 0.979; SRMR = 0.042; chi-square/df = 22.867/16, *p* = .117). Age (*β* = − 0.182, *p* < .001) was found to act as a moderator between discrimination and psychological distress.

**Conclusion:**

The findings reveal that resilience significantly reduces psychological distress among rural Black women, in the context of the adverse effects of discrimination on well-being. Aging appears to exert a protective influence on mental health outcomes, in the presence of discrimination, perhaps as related to generational differences in experiences, perceptions, and coping. In alignment with the advancement towards racial equity, reducing discrimination and fostering process-oriented resilience that is multilevel and multidimensional emerge as potential strategies to lessen the negative impacts on psychological well-being.

## Introduction

Black populations in the USA face historic, systemic racism, which has been identified as an influential determinant of health [1]. Racism, according to a systematic review and meta-analysis, is associated with negative physical and mental health outcomes [1]. Within the USA, Black populations experienced 1.63 million excess deaths between 1999 and 2020 [2]. In addition, Black populations experience a variety of unique yet compounding vulnerabilities as related to additional identities and contexts (e.g., age, SES, pregnancy, geography). Health disparities can be understood as a result of structural racism (i.e., distal, upstream factors within institutions, policies, and society at large that differentially oppress or advantage populations) [3]. Racialized police violence is one pertinent example of structural racism [3]. Although activism against police brutality had been ongoing, the Black Lives Matter movement gained national attention in 2020, with accompanying protests and action across the USA, in response to the murders of Ahmaud Arbery, Breonna Taylor, and George Floyd and the broader systems that oppress Black populations [4]. Within the movement, there emerged the Say Her Name Campaign to draw attention to the differential, and often overlooked, experiences of Black women (i.e., cisgender and transgender women) [5]. As demonstrated by the need for the Say Her Name Campaign, individuals have a variety of identities and contexts that may coexist and interact within overarching systems of oppression.

*Multiple Jeopardy*, a term coined by Deborah King, illustrates the multiplicative effects of various identities or vulnerabilities (e.g., race/ethnicity, gender, socioeconomic status) that interact simultaneously, particularly as experienced by Black women [6]. Relatedly, *intersectionality*, a term coined by Kimberlé Crenshaw, delineates the multidimensionality of identities and the ways in which they intersect and result in compounding experiences of discrimination and forms of oppression [7]. These frameworks, when considered alongside the cumulative disadvantage theory, contextualize the accumulating nature of experiences of discrimination and oppression over the life course [8]. Within individuals, accumulated disadvantage, as related to the multiple jeopardy of oppression experienced across a variety of vulnerable identities, is associated with weathering, demonstrating the impact of structural inequities in producing and accelerating health disparities [8]. Within these schools of thought, Black women are a priority given their multiple, intersecting vulnerabilities associated with their interdependent identities such as gender, race, ethnicity, age, and geography [9].

Discrimination (i.e., prejudicial or negatively differential treatment related to personal characteristics), based upon an individual’s social identities (e.g., race, ethnicity, gender, sexual orientation), has been found to adversely impact mental health and is a primary driver of racial health disparities [10]. From an intersectional perspective, individuals are comprised of a variety of intersecting social identities and may simultaneously experience discrimination and overlapping systems of oppression. Discrimination, including racial discrimination, has been found to have adverse effects on mental health and to be inversely associated with mental health assets (e.g., resilience, quality of life, self-esteem, life satisfaction) [10, 11]. Due to their role in alleviating the effects of discrimination, mental health assets have received increased attention [10, 11]. A scoping review of resilience among Black or African American women contextualizes traditional definitions of resilience (e.g., the ability to bounce back in the face of adversity, coping with stress) within the persistent adversity of systemic racism and highlights the need to conceptualize resilience as a multidimensional process (i.e., including the social determinants of health, spirituality and religion, strength, survival, active coping, and social support) [12].

Given the mixed findings and associations established within the literature, there remain knowledge gaps and calls for further research that investigates the experiences and impacts of discrimination by sex and/or gender due to documented variation [11]. Further, the resilience literature has been described as failing to prioritize and consider resilience within the contexts unique to vulnerabilized subpopulations such as Black, Hispanic, and Latina women [13, 14]. Therefore, this study aims to (1) explore if resilience mediates the negative impacts of discrimination on psychological well-being among Black women living in rural South Carolina in the post-COVID-19-pandemic era and (2) if certain demographics may moderate the association between discrimination and psychological well-being. Based upon literature that has identified generational differences in perceptions of mental health challenges among Black women, and as informed by the life course perspective, it is hypothesized that age will moderate the impact of discrimination on psychological distress with a lower impact among older Black women, compared to their younger counterparts [15, 16].

## Methods

### Study Design

Participants were sampled using a cluster sampling approach among 23 counties across South Carolina. Within these counties, those eligible to participate were aged 18 years or older, identified as Black, identified as a woman, and lived in rural South Carolina during the COVID-19 pandemic (i.e., 2020–2023) [17]. Participants (*n* = 336) were recruited between January and February of 2024. Through a partnership with a statewide community health worker (CHW) organization, community health workers received training to recruit participants and administer the study survey. All participating CHWs (*n* = 28) were referred or self-selected, based on their interest in community engaged research, and attended multiple online training sessions with CHW leadership and the research team. Topics covered in the training included the project purpose, foundations of community-based research, privacy and confidentiality, survey methodology in rural settings, ethics, equity, and data quality and validity. Additionally, the CHWs reviewed the study survey and engaged in meetings with the full team where all comments, questions, and concerns were discussed. The feedback from the CHWs led to the refinement of the content and language of the survey to ensure its applicability to the target study population. CHWs provided valuable insight through their pilot testing of the questionnaire, prior to its dissemination, due to their personally disclosed identities that mirror those of the target study population as well as their familiarity with their local, rural communities.

### Data Collection

Trained CHWs were responsible for both participant recruitment and data collection within their communities. Participants completed a self-administered online survey on the HIPAA compliant platform REDCap [18, 19]. All survey data were automatically uploaded to the dual-factor authentication protected REDCap server at the University of South Carolina. During the survey, a CHW was present to provide any needed clarification or technology support. In preparation for potential internet access issues, all CHWs were equipped with a paper copy of the survey, with protocols in place for the participant to seal their responses and CHWs to deliver them to the research team; however, no participants faced technical issues to require this action. The survey was in English and required 45 to 60 minutes to complete. The first page of the survey included the invitation to participate which briefly described the study purpose, estimated time required to participate, the voluntary nature of participation, confidentiality, the intent to publish or present deidentified study findings, and the research team and office of research compliance’s contact information, for any questions. Participants indicated their consent to participate by continuing to the next page of the survey. For their time and effort in recruitment and data collection, CHWs received a $125 USD incentive for completing the training and $25 USD for each participant who was recruited and completed the survey. For their time and effort completing the survey, participants received a $25 USD incentive upon survey completion. The study activities were reviewed and approved by the Institutional Review Board at the University of South Carolina.

### Measures

#### Discrimination

An average discrimination score was calculated based on participant responses to the Everyday Discrimination Scale (EDS) [20]. The possible range of average scores on the EDS spanned from 1 to 6, with a higher score indicating more frequent experiences of discrimination. The EDS is comprised of nine items that ask participants to indicate the level to which they are discriminated against in their daily life. Examples of experiences of discrimination captured within the scale included being treated or perceived differently than others and experiencing name-calling or harassment. The response options were (1) never, (2) less than once a year, (3) a few times a year, (4) a few times a month, (5) at least once a week, and (6) almost every day. Following the EDS, participants were asked to check all that apply to indicate the sources of the discrimination they experience (i.e., friends and family, strangers, community members, neighbors, co-workers, healthcare providers) and the social identities associated with the experienced discrimination (e.g., age, gender, height, weight, race, ancestry or nationality, citizenship or immigration status, education, income). The EDS demonstrated adequate internal consistency (*α* = 0.75, CI = 0.69, 0.81).

#### Resilience

An average resilience score was calculated based on participant responses to the Brief Resilience Scale [21]. The average resilience scores possible ranged from 1 to 5, with a higher score indicating greater resilience. The response options were (1) strongly disagree, (2) disagree, (3) neutral, (4) agree, and (5) strongly agree. Items were reverse coded as necessary. The Brief Resilience Scale measured participants’ perceptions of their ability to bounce back and persevere through difficult or stressful times. The Brief Resilience Scale demonstrated adequate internal consistency (*α* = 0.74, CI = 0.67, 0.80).

#### Psychological Distress

Psychological distress was operationalized as a composite score of three different scales which measured anxiety (GAD-7), depression (PHQ-9), and post-traumatic stress disorder (PTSD, PC-PTSD-5) [22–24]. The GAD-7 measured participants’ self-reported frequency of anxiety, stress, and worry with response options ranging from (0) not at all to (3) nearly every day. The PHQ-9 captured experiences indicative of depression such as decreased interest, hopelessness, limited energy, and difficulty concentrating. Response options to the PHQ-9 ranged from (0) not at all to (3) nearly every day. The PC-PTSD-5 measured experiences of PTSD such as feeling numb, on guard, and guilty and having nightmares with the binary response options of (1) yes or (0) no.

#### Demographics

The primary demographics of age, education, and income were collected. Participant age in years was treated as a continuous variable. Education was treated as a continuous variable with response options of (1) less than a high school diploma; (2) high school diploma or equivalent; (3) some college, but no degree; (4) associate degree; (5) bachelor’s degree; and (6) post-graduate degree or above. Participants indicated their total annual household income with response options ranging between less than $10,000 USD and $200,000 USD or more. Due to a significant number of participants that opted out of providing their annual household income (i.e., prefer not to answer), the variable of income was not included as a covariate in the model. Therefore, age and education were included as continuous covariates in the final model.

### Data Analysis

The data were cleaned prior to analysis. As the sample had a large age range (i.e., participants reporting an age over 80), age was restricted from the 1st to the 99th percentile, removing influential outliers (*n*=8). The Mahalanobis approach in R was used to detect outliers by calculating each datapoint’s distance from the mean of the multivariate distribution. This approach was applied to demographic and outcome variables to detect responses which deviated from the patterns of the majority. Outliers were removed for the primary variables in the study including discrimination, resilience, anxiety, depression, and PTSD (*n*=28). In total, 36 outliers were detected and excluded from the analysis. Missing responses were addressed through mean imputation. Correlations were assessed using Pearson’s correlation coefficients. Structural equation modeling was performed to examine the hypothesized mediation and moderation model. The maximum likelihood robust (MLR) estimator was used to account for non-normality. The kurtosis and skewness of all measurements were assessed and deemed to be within a tolerable non-normality range (i.e., kurtosis between − 7 and 7, skewness between − 2 and 2). Analyses were conducted using the SEM function in the Lavaan package in R version 4.2.0. The model tested an explanatory mechanism with resilience as a mediator and age as a moderator. The final model’s goodness of fit was tested through multiple indices: the root mean square error of approximation (RMSEA), the comparative fit index (CFI), the Tucker–Lewis index (TLI), and the standardized root mean squared residual (SRMR). The thresholds of these indices to indicate a good model fit are as follows: 0.05 (good) to 0.08 (adequate) for RMSEA, 0.90 (adequate) to 0.95 (good) for CFI, 0.90 (adequate) to 0.95 (good) for TLI, and 0.08 (good) for SRMR [25]. Higher values of TLI and CFI and lower values of RMSEA and SRMR suggest a better fit of the data. Delta *z* scores examined the indirect effect of discrimination on psychological distress through resilience after controlling for demographic characteristics.

## Results

The full sample of participants included 336 individuals, of whom 300 remained after data cleaning to remove outliers. In alignment with the study aims and inclusion criteria, all participants were women who identified as Black (*n* = 300, 100%). As shown in Table 1, the mean age of the sample was 48.6 years (SD = 16.8). A quarter of participants (26.3%) held a high school diploma or equivalent and almost half of participants (46.6%) indicated an annual household income of $35,000 USD or less. Participants reported an average score of 1.73 (SD = 0.86) on the Everyday Discrimination Scale (EDS). Participants primarily indicated the discrimination experienced as related to their race (53.7%, *n* = 161), age (22.0%, *n* = 66), and/or gender (18.3%, *n* = 55). The overall average score of resilience was 3.63 (SD = 0.72). The composite score of psychological distress included the sum scores of the measures of anxiety (mean = 4.04, SD = 4.21, *α* = 0.93, CI = 0.91, 0.95), depression (mean = 4.45, SD = 4.98, *α* = 0.93, CI = 0.91, 0.94), and PTSD (mean = 0.86, SD = 1.40, *α* = 0.80, CI = 0.74, 0.85).

**Table 1:** Participant demographics (*n* = 300)

Variable	Overall	Aged < 50	Aged 50 +	Range
	*n* (%) or mean (SD)			
**Race/ethnicity** **Black**	300 (100%)	147 (100%)	153 (100%)	
**Age**	48.6 (16.8)	33.9 (9.07)	62.7 (8.38)	
**Gender** **Women**	300 (100%)	147 (100%)	153 (100%)	
**Education** **Less than high school** **High school or equivalent** **Some college** **Associates** **Bachelors** **Post-graduate or above** **Prefer not to answer**	11 (3.7%) 79 (26.3%) 66 (22.0%) 49 (16.3%) 53 (17.7%) 33 (11.0%) 9 (3.0%)	2 (1.4%) 38 (25.9%) 37 (25.2%) 25 (17.0%) 29 (19.7%) 13 (8.8%) 3 (2.0%)	9 (5.9%) 41 (26.8%) 29 (19.0%) 24 (15.7%) 24 (15.7%) 20 (13.1%) 6 (3.9%)	
**Annual income** **Less than $10,000** **$10,000–$24,999** **$25,000–$34,999** **$35,000–$49,999** **$50,000–$74,999** **$75,000–$99,999** **$100,000–$149,999** **$150,000–$199,999** **Prefer not to answer**	24 (8.0%) 55 (18.3%) 61 (20.3%) 43 (14.3%) 35 (11.7%) 24 (8.0%) 8 (2.7%) 3 (1.0%) 47 (15.7%)	14 (9.5%) 24 (16.3%) 32 (21.8%) 25 (17.0%) 19 (12.9%) 12 (8.2%) 6 (4.1%) 1 (0.7%) 14 (9.5%)	10 (6.5%) 31 (20.3%) 29 (19.0%) 18 (11.8%) 16 (10.5%) 12 (7.8%) 2 (1.3%) 2 (1.3%) 33 (21.6%)	
**Discrimination**	1.73 (0.86)	1.75 (0.81)	1.71 (0.91)	1–6
**Psychological distress** **Anxiety** **Depression** **PTSD**	4.04 (4.21) 4.45 (4.98) 0.86 (1.40)	4.91 (4.32) 5.46 (5.54) 1.14 (1.6)	3.2 (3.69) 3.47 (3.7) 0.59 (1.1)	0–21 0–27 0–5
**Resilience**	3.63 (0.72)	3.55 (0.7)	3.72 (0.72)	1–5

The correlations between the primary measures of the study are shown in Table 2. Depression was positively associated with anxiety (0.75, *p* < 0.001), PTSD (0.55, *p* < 0.001), and discrimination (0.39, *p* < 0.001) and was negatively associated with resilience (− 0.41, *p* < 0.001). Anxiety was positively associated with PTSD (0.56, *p* < 0.001) and discrimination (0.34, *p* < 0.001) and was negatively associated with resilience (− 0.37, *p* < 0.001). PTSD was positively associated with discrimination (0.27, *p* < 0.001) and was negatively associated with resilience (− 0.34, *p* < 0.001).

**Table 2:** Correlation matrix of primary measures

Variables	Range	Depression	Anxiety	PTSD	Discrimination	Resilience
Depression	0–27	1				
Anxiety	0–21	0.75***	1			
PTSD	0–5	0.55***	0.56***	1		
Discrimination	1–6	0.39***	0.34***	0.27***	1	
Resilience	1–5	− 0.41***	− 0.37***	− 0.34***	− 0.11†	1

*p < 0.05, **p < 0.01, ***p < 0.001; †*p* = 0.062

**Fig. 1:**
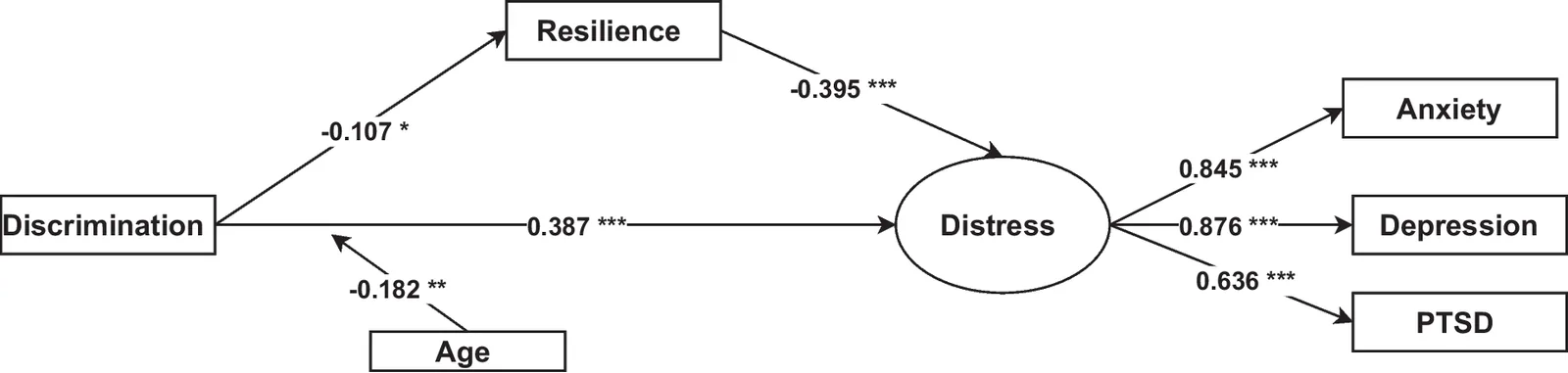
SEM model of discrimination, resilience, and psychological distress among Black women living in rural South Carolina (*n* = 300)

Within the SEM, values represent standardized factor loadings and standardized correlation estimates. The model had one fixed indicator loading (i.e., anxiety to 1) for the latent factor, psychological distress, to ensure proper model identification. The goodness-of-fit indicators suggest a good fit of the model to the data (RMSEA = 0.038; CFI = 0.986; TLI = 0.979; SRMR = 0.042; chi-square/df = 22.867/16, *p* = 0.117). As shown in Fig. 1 and Table 3, discrimination was directly, negatively associated with resilience (*β* = − 0.107, *p* < 0.05) and resilience was directly, negatively associated with distress (*β* = − 0.395, *p* < 0.001). The direct path between discrimination and distress was also significant, with the presence of resilience in the model (*β* = 0.387, *p* < 0.001). In terms of potential confounders, age and education were not significantly associated with resilience or psychological distress. The indirect effect of discrimination on distress was tested using the delta *z* score. Results showed that, through resilience, discrimination had a positive, indirect effect on distress (*β* = 0.042, *z* = 2.004, *p* < 0.05). The significant indirect and direct effects of discrimination on distress suggest partial mediation. Age (*β* = − 0.182, *p* = 0.003) acted as a moderator between discrimination and psychological distress, potentially indicating the protective influence of aging on negative mental health outcomes (Tables 4 and 5).

**Table 3:** Decomposition of the effects of discrimination on psychological distress

	***β***	**95%* CI***	***p* value**
**Direct effect**			
Discrimination → psychological distress	0.387	(0.280, 0.490)	0.000
Discrimination → resilience	− 0.107	(− 0.21, − 0.005)	0.04
Resilience → psychological distress	− 0.395	(− 0.504, − 0.285)	0.000
**Indirect effect**			
Discrimination → resilience → distress	0.042	(0.001, 0.083)	0.045

**Table 4:** Main and interactive effects of discrimination and age on psychological distress

	***β***	**95%* CI***	***p* value**
**Main effect**			
Discrimination → psychological distress	0.387	(0.280, 0.490)	0.000
Age	− 0.150	(− 0.247, − 0.052)	0.003
**Interactive effect**			
Discrimination × age	− 0.182	(− 0.3, − 0.064)	0.003

**Table 5:** Conditional direct and total effects of discrimination on psychological distress through age

	***β***	**95%* CI***	***p* value**
**Direct effect**			
Young age Discrimination → psychological distress	0.569	(0.385, 0.753)	0.000
Older age Discrimination → psychological distress	0.205	(0.072, 0.338)	0.002
Total effect			
Young age Discrimination → psychological distress	0.611	(0.428, 0.794)	0.000
Older age Discrimination → psychological distress	0.247	(0.108, 0.386)	0.000

## Discussion

Within the present study, discrimination was negatively associated with resilience and positively associated with psychological distress, measured as anxiety, depression, and PTSD. Discrimination had a positive direct effect on psychological distress and a negative direct effect on resilience. Resilience was found to mediate the impact of discrimination on psychological distress through a positive indirect effect. Overall, the findings of the present study provide support for the established role of resilience in coping with the psychological distress experienced as a result of discrimination but extend these findings to a subpopulation of Black women living rurally in the deep south in the post-pandemic era [12]. These findings also contribute further evidence of age as a buffer between exposure to racism and negative psychological outcomes, demonstrated in this study as the protective nature of aging in coping with discrimination among Black women who live rurally [26].

In this study, resilience was conceptualized as a process through which individuals are able to effectively adapt and respond to hardship in challenging times or in the wake of adversity [21]. Where discrimination was found to have a negative effect on psychological well-being, resilience was found to have a positive indirect effect. The importance of resilience as a protective factor is underscored by its potential to buffer the harmful effects of discrimination on psychological well-being (i.e., depression) and subsequent acceleration of biological aging among those facing long-term social inequality [27]. Within the literature, coping strategies relied upon by Black women to foster resilience, within a system that is entrenched in structural racism, include spirituality, social support, redefining Black womanhood, self-expression within safe spaces, and resistance [12, 28]. Despite their protective characteristics, individual psychological resources alone are not sufficient to avoid the negative effects of chronic stress on health (i.e., weathering) [29]. Further, resilience may reflect the double-edged sword of the *superwoman schema* phenomenon where Black women face societal expectations to embody certain attributes (i.e., obligation to manifest strength, obligation to suppress emotions, resistance to being vulnerable or dependent, determination to succeed despite limited resources, obligation to help others) despite facing perpetual, systemic racism, which can act as both an asset and a vulnerability to health status [30, 31]. Therefore, although resilience plays an important, protective role in everyday life, there remains a need to address distal, systemic causes and perpetuations of discrimination and inequity.

As corroborated in the present study, older age was found to buffer, or moderate, the effects of discrimination on psychological well-being [26]. Within the literature, younger Black women have been found to experience a higher severity of anxiety as a result of discrimination, in comparison to their older counterparts [26]. The moderating effect of age on psychological well-being exists simultaneously alongside the presence of weathering and accumulated disadvantage that compound into older adulthood. Generational differences may exist in coping with discrimination and its effects on mental health that may be related to differential experiences and perceptions of overt (e.g., explicit prejudice) and covert (e.g., passive prejudice, microaggressions) racism over time. The findings of the present study align with previous literature that has identified generational differences in perceptions of mental health challenges among Black women [15, 16]. For example, the literature has described that perceptions of mental health differ generationally, alongside norms to avoid discussions of mental health and expectations that may be dismissive of mental health challenges (e.g., expectation of the strong Black woman, superwoman schema) [15]. Similarly, another qualitative study found generational differences in mental health diagnosis and trauma, where younger populations described their experiences being raised by and living with older generations who had unaddressed mental health challenges [16]. Alternatively, there is literature that has found that some mental health challenges (i.e., major depressive disorder) increase with age [32]. The present findings contribute to the emergent literature, but the mixed findings, within the literature, call for additional research.

The present study is characterized by several strengths and limitations. A strength of this work is the focus on the experiences of Black women who are often not included in research (e.g., rural residents, older age). On the other hand, the specificity of the study population may limit the generalizability of the results beyond a deep south context. An additional limitation of this work is the cross-sectional design. Further, although the kurtosis and skewness were within a tolerable non-normality range, there are potential floor effects among the included measures. The study may also underestimate psychological distress, while potentially overestimating resilience, due to the reliance on self-report measures that may be susceptible to the social-desirability bias.

In light of the present findings, there remains a need for further research. First, longitudinal data are needed to further evaluate resilience from a dynamic, multidimensional perspective. Second, intersectionality should be incorporated into measurement [33] and analysis [34, 35]. For example, as variation in EDS question interpretation has been identified within the literature, future studies should rely also on intersectional measures of discrimination and could leverage cognitive interviewing techniques to explore, in-depth, participants’ interpretations and frames [36]. Finally, considering the double-edged sword of the superwoman schema, further research should consider the potentially damaging effects of fixating on individual resilience. Given the findings of the present work, there is a call for public health efforts to structurally address systemic perpetuations of racism and discrimination. In addition to addressing systemic racism and discrimination, fostering resilience from a multidimensional, multilevel process perspective emerges as a potential approach to addressing the negative effects of discrimination on the well-being of rural, Black women.

## Data Availability

The data that support the findings of the present study are available, upon reasonable request, from the corresponding author.
